# Enhancing Gluten-Free Bread Production: Impact of Hydroxypropyl Methylcellulose, Psyllium Husk Fiber, and Xanthan Gum on Dough Characteristics and Bread Quality

**DOI:** 10.3390/foods13111691

**Published:** 2024-05-28

**Authors:** Ramón Torres-Pérez, Elena Martínez-García, Marta Maravilla Siguero-Tudela, Purificación García-Segovia, Javier Martínez-Monzó, Marta Igual

**Affiliations:** i-Food, Instituto Universitario de Ingeniería de Alimentos-FoodUPV, Universitat Politècnica de València, Camino de Vera s/n, 46022 València, Spain; ramon@sinblat.es (R.T.-P.); elenamartinezgarcia134@gmail.com (E.M.-G.); martamaravilla00@gmail.com (M.M.S.-T.); pugarse@tal.upv.es (P.G.-S.); xmartine@tal.upv.es (J.M.-M.)

**Keywords:** gluten free, bread, hydroxypropyl methylcellulose (HPMC), psyllium, xanthan gum, texture profile analysis (TPA)

## Abstract

The demand for gluten-free products has increased due to improved diagnoses and awareness of gluten-related issues. This study investigated the effect of HPMC, psyllium, and xanthan gum in gluten-free bread formulations. Three tests were conducted, varying the amount of these ingredients: in the first formulation, the amount of HPMC was increased to 4.4 g/100 g of flour and starch; in the second, psyllium husk fiber was increased to 13.2 g/100 g of flour and starch; and in the third formulation, xanthan gum was removed. Differences were observed among the formulations: increasing HPMC reduced extrusion force without affecting bread quality; adding psyllium increased dough elasticity but also crumb gumminess and crust hardness. Eliminating xanthan gum altered dough rheology, resulting in a softer and less gummy crumb, and a less reddish color in the final bread.

## 1. Introduction

In recent years, an increase in consumer demand for gluten-free products has been observed [1,2]. This notable rise in the number of individuals following a gluten-free diet can be attributed to improved diagnoses and greater awareness of gluten allergy, intolerance, and sensitivity [3,4,5]. Additionally, some consumers believe that gluten-free foods are healthier than their gluten-containing counterparts [6]. All of this has led to an increase in the production of gluten-free bread, despite it being one of the most challenging products to produce [7].

Currently, the production of gluten-free bread is carried out on lines designed for gluten-containing doughs. However, these processes are not always directly transferable to gluten-free product production [8]. These lines are designed to sheet and portion the dough or subject it to extrusion to provide pieces of dough with a constant volume [9].

Gluten forms a unique network that creates a viscoelastic dough capable of retaining gases [10]. In addition to trapping fermentation gases, the gluten network plays a crucial role in creating the cellular structure of the crumb, resulting in a distinctive texture and flavor not found in other baked goods [11]. However, developing gluten-like characteristics in gluten-free doughs can be challenging.

Currently, hydrocolloids such as hydroxypropyl methylcellulose (HPMC), xanthan gum, psyllium husk fiber, and guar gum are the most commonly used ingredients to aid in this process [12]. Psyllium husk fiber is a soluble dietary fiber that offers numerous physiological benefits, including blood sugar control, cholesterol reduction, the prevention of constipation, and a lower risk of cardiovascular diseases [13]. Studies like that of Zandonadi et al. [14] have observed that gluten-free products formulated with psyllium are well received sensorially, both by celiac and non-celiac individuals. It has also been found to improve dough handling during processing [15], although careful adjustment of dosages and dough hydration is necessary. Ren et al. [16] and Mancebo et al. [17] pointed out that the inclusion of psyllium in gluten-free bread formulation must be carefully adjusted to fully benefit from its functionalities, as an inappropriate combination of psyllium and hydration levels can lead to bread with a reduced volume and excessively hard crumb.

HPMC, derived from modified cellulose, has a high water-binding capacity due to its hydrophilic nature, as described by Djordjević et al. [18]. Furthermore, the hydrophobic groups present allow for structuring through network formation, resulting in increased development, gas retention capacity, and specific volume in gluten-free bread. This effect of improving specific volume has an impact on the tenderness of the bread [19]. Xanthan gum is one of the most commonly used hydrocolloids in gluten-free bread formulations [7,20]. However, Hager et al. [21] observed that the addition of xanthan gum had a negative linear effect on bread volume.

This study was designed to investigate the impact of increasing hydroxypropylmethylcellulose (HPMC), psyllium husk fiber, and xanthan gum in gluten-free doughs, aiming to understand their behavior on production lines and their influence on the resulting bread’s characteristics. A control formulation (CF) containing the three mentioned hydrocolloids was used, as it was observed that gluten-free bread sold in Spanish supermarkets contained this hydrocolloid mixture. Three tests were conducted, starting from a control recipe. In the first formulation, the amount of HPMC was increased to 4.4 g/100 g of flour and starch; in the second, psyllium husk fiber was increased to 13.2 g/100 g of flour and starch; and in the third formulation, xanthan gum was eliminated. Both psyllium and xanthan gum exhibit similar rheological behaviors, as both are responsible for weak gelling properties, Belorio et al. [22]. Therefore, the impact of their elimination from the formula was investigated.

The amount of water was adjusted in the formulations to compensate for the hydrocolloids’ effect on water absorption. Rheological analyses were conducted to understand the effect of the different formulations on dough elasticity and viscosity, compression–extrusion tests to measure dough hardness during extrusion, and physicochemical and color analyses on the dough. Subsequently, physicochemical analyses of the produced bread, a crust and crumb color analysis, a texture profile analysis, crust puncture tests, and image analyses of the slices were performed to quantify the number and size of the cells.

## 2. Materials and Methods

### 2.1. Formulations

In this section, the ingredients used in the gluten-free bread formulation are described, as well as the bread-making process.

#### 2.1.1. Ingredients

Gluten-free bread was made with corn starch (Roquette, Benifaió, Spain), rice flour (Arrocerías San Cristóbal, Sollana, Spain), tapioca starch (Vicorquimia, Badalona, Spain), sucrose (Aceites La Canal, Chella, Spain), sunflower oil (Aceites La Canal, Chella, Spain), rice syrup (Ferrer Alimentación, Barcelona, Spain), compressed yeast (Lesaffre Iberica, Valladolid, Spain), and EGM gluten-free bread mix (Sinblat Alimentación Saludable, Foios, Spain), which was made of salt, inactive sourdough, sodium bicarbonate, monocalcic phosphate, pea protein, bamboo fiber, potassium sorbate, calcic propionate, natural flavors, and enzymes.

Gluten replacers were hydroxypropyl methylcellulose (HPMC) Vivapur K4M (Rettenmaier Ibérica, Barcelona, Spain), xanthan gum (Brenntag, Dos Hermanas, Spain), and psyllium husk fiber (Barcelonesa, Cornellà de Llobregat, Spain).

#### 2.1.2. Gluten-Free Breadmaking

A gluten-free bread recipe was composed as follows (per 100 g of flour and starch): 91.2 g of maize starch, 5.5 g of rice flour, 3.3 g of tapioca starch, 17.9 g of EGM gluten-free bread mix, 5 g of sucrose, 26.2 g of sunflower oil, 7.1 g of rice syrup, and 7.7 g of compressed yeast. HPMC, xanthan gum, psyllium, and tap water were added in varying amounts to each formulation. Differences in composition can be observed in Table 1.

Three tests were conducted, starting from a control recipe. In the first formulation, the amount of HPMC was increased to 4.4 g/100 g of flour and starch; in the second, psyllium husk fiber was increased to 13.2 g/100 g of flour and starch; and in the third formulation, xanthan gum was eliminated.

The amount of water was determined based on a subjective visual evaluation to achieve similar dough consistencies across all formulations. In the case of HPMC, it was observed that for each additional gram, it was necessary to compensate with 5.4 g of water. This value is similar to the calculations made by Kim et al. [23], where a 5% increase in water was added for each gram of HPMC added to whole wheat flour. For psyllium, 4 g of water was added, and for xanthan gum, 3.5 g of water was added for each gram of hydrocolloid. In the study conducted by Belorio et al. [22], it was observed that the water-holding capacity of xanthan and psyllium was 3.64% and 3.42%, respectively.

All the ingredients were mixed using a Sigma Aeromix mixer (Sigma SRL, Torbole Casaglia, Italy) with a hook tool, all mixed at speed one for 15 min. The dough was divided into pieces weighing 440 g and shaped by hand before being put into a Teflon-coated metal mold measuring 9.9 × 19.1 × 6.8 cm. The molds were fermented in a Bauuman fermenter (Bauuman Tech. SL, Valencia, Spain) at 29 °C and 80% relative humidity until they reached three times their initial volume. After fermentation, the bread pieces were placed in an oven (FOX 10T-LFRC, Logiudice Forni SRL Unipersonale, Arcole, Italy) and baked at 210 °C for 2 min, followed by 38 min at 180 °C. Once baked, the breads were removed from the mold and allowed to cool in a room at 25 °C for 2 h. Afterward, loaves were sliced, packed in polypropylene bags, and stored under controlled conditions (22−25 °C, 50–70% RH). Bread samples were analyzed 48 h after production.

### 2.2. Analysis

#### 2.2.1. Rheological Properties

The rheology of the dough was characterized using a rheometer (Kinexus Pro+, Malvern Instruments, Worcestershire, UK). Oscillatory tests were conducted to assess the rheological properties of the gluten-free bread doughs. Oscillatory testing is employed for analyzing the structure and determining the mechanical properties (viscoelasticity) of a material in its molten state. This method preserves the structure of the material and deformation occurs at small strain rates in an oscillatory manner. The following parameters were employed for the oscillatory tests: a plate size of 40 mm, gap of 2 mm, frequency range of 0.1–10 Hz, and strain of 0.5%. An amplitude sweep was performed to determine the linear viscoelastic region. The initial shear stress ranged from 0.1% to 100% at the end at a 1 Hz frequency. Tests were performed in triplicate for each dough formula, and all experiments were conducted at 20 °C.

#### 2.2.2. Extrusion Analysis

The extrusion analysis was conducted using a TA-XT Plus texture analyzer equipped with a load cell of 50 kg (Stable Micro Systems, Surrey, UK). To assess the texture of the dough, a compression–extrusion test was performed, involving the application of force to a product until it flowed through an orifice (or opening). The product was compressed and extruded through this opening, providing the average extrusion force (N), and area (N·s). A Forward Extrusion Cell was used for the test, with a base disc with a 5 mm outlet diameter. The test parameters were a pre-test speed of 1 mm/s, test speed of 2 mm/s, post-test speed of 10 mm/s, and displacement of 20 mm. This analysis was conducted on four samples per formulation.

#### 2.2.3. Physicochemical Analysis

Physicochemical analyses were conducted on both the dough and the bread obtained from each formulation. The crust and crumb of the bread were analyzed separately.

The water content of the samples (x_w_) was determined using a Vaciotem-T vacuum oven (J.P. Selecta, S.A., Barcelona, Spain). Samples were pre-dried for 24 h before undergoing vacuum oven drying for 48 h. Moisture content in dough and bread determinations were conducted in triplicate.

Water activity (aw) was assessed using an AquaLab PRE (Decagon Services, Inc., Pullman, WA, USA). Following conditioning and calibration, water activity measurements were conducted in triplicate on both the crust and crumb of all bread samples produced.

The pH of bread was measured using a Crison Basic 20+ pH meter equipped with puncture electrodes designed for solid products (Crison Instruments S.A., Barcelona, Spain). The pH measurement was conducted solely on the crumb of the bread. Six measurements were made for each bread.

#### 2.2.4. Color Analysis

The color analysis was performed on both the dough and the crust and crumb of the bread obtained from each formulation. It was measured using a colorimeter (CR-400, Konica Minolta, Tokyo, Japan). The device was calibrated using the standard illuminant D65 and a 10° observer. Readings were displayed as color parameters a*, b*, L*, and ΔE according to the CIELAB color measurement system. The a* value represents a measure of greenness ranging from −100 to +100 (redness), the b* value ranges from −100 (blue) to +100 (yellow), and the L* value indicates the measure of lightness and ranges from 0 (black) to 100 (white). The formula used to calculate the ΔE value is given below:

(1) $$\Delta E=\sqrt{{\left(L-L_{0}\right)}^{2}+{\left(a-a_{0}\right)}^{2}+{\left(b-b_{0}\right)}^{2}}$$

where L, a, and b are the color parameters of the measured sample; L_0_, a_0_, and b_0_ are the parameters of control bread, named as Fc. The color of the dough was determined twelve times at twelve different points for each formulation.

As for the crust and crumb, it was determined at least six times at six points on different slices of all the bread made.

#### 2.2.5. Texture Analysis

The texture analysis was conducted using a TA-XT Plus texture analyzer equipped with a load cell of 50 kg (Stable Micro Systems, Surrey, UK). To assess the crumb texture, a texture profile analysis (TPA) was performed using eight central slices, each 5 mm thick, from three loaves of each formulation. A P/75 compression plate was used, with a test speed of 5 mm/s and a deformation of 50%. Recorded parameters included hardness, adhesiveness, cohesiveness, and gumminess. TPA was carried out on eight samples per formulation.

#### 2.2.6. Image Analysis

Digital photographs were taken from 5 mm thick slices. The slices were scanned using an Epson SX420 scanner (Seiko Epson Corporation, Tokyo, Japan). The image analysis was performed using Image J software (ImageJ, NIH, Bethesda, MD, USA). The number of cells and the average area of the cells (in mm^2^) were determined. Area values ranging from 0.15 to 10.00 mm^2^ were established as the lower and upper limits, respectively, for cells to be considered by the software [24].

#### 2.2.7. Statistical Analysis

The data were analyzed using the analysis of variance (ANOVA) technique, with the Fisher LSD test at a 95% confidence level (*p* < 0.05). These statistical analyses were carried out using Statgraphics Centurion XVII v204 8 (Statgraphics Technologies, Inc., The Plains, VA, USA).

## 3. Results and Discussion

### 3.1. Dough Rheology Properties

**Figure 1:**
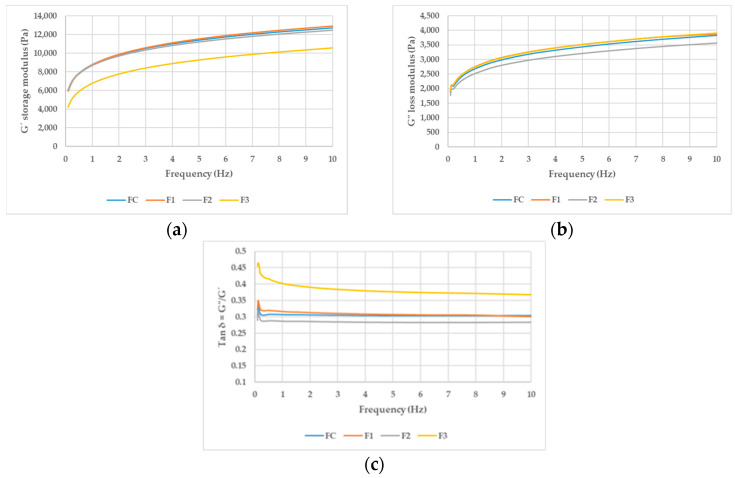
(**a**) G′ storage modulus (Pa); (**b**) G″ loss modulus (Pa); (**c**) Tan δ = G″/G′. FC: control formula. F1: HPMC increase. F2: psyllium increase. F3: xanthan gum removal.

As shown in Figure 1 and Table 2, all values for the storage modulus (G′) were higher than those for the loss modulus (G″). Consequently, the values of Tan δ in all formulations were less than 1. This indicates that, in all formulations, the dough exhibited predominantly elastic behavior. A similar effect was observed by Farahnaky et al. [25], who noted that psyllium is associated with a weak gel, characterized by G′ always being greater than G″, and both moduli being almost parallel to each other across different frequencies. In that study, it was observed that the values of G′ and G″ increased with an increase in the amount of psyllium. This phenomenon has also been reported in studies on the rheology of gluten-free bread formulations, such as those conducted by Ren et al. [16], which used psyllium and methylcellulose, and Mancebo et al. [17], where HPMC and psyllium were incorporated. Similarly, in the works of Lazaridou et al. [26] and Demirkesen et al. [27], which included xanthan gum, comparable effects were observed.

Figure 1 shows the average curves for each formula, grouped according to the parameter (G′, G″, and Tan δ).

In Table 2, it can be observed that the increase in HPMC in formulation F1 compared to FC did not show significant differences in the rheology of the dough. In studies conducted by Hussain et al. [28], various amounts of HPMC were added to deionized water, and it was observed that the loss modulus (G″) was greater than the storage modulus (G′), indicating a predominance of the viscous component. Rosell and Foegeding [29] observed an elastic behavior, with G′ being higher than G″ when HPMC was added to a mixture of gluten and distilled water. Sivaramakrishnan et al. [30] noted that rice flour exhibited elastic behavior, but its rheology was altered by the addition of HPMC, with the effect being more pronounced when the amount of HPMC was increased from 1.5% to 4.5%. Djordjević et al. [18] observed a decrease in the flow behavior index with the increase in HPMC, resulting in more pseudoplastic doughs.

In the results obtained in our study, the increase in HPMC showed no significant differences compared to FC. It is important to note that the hydration level is a key factor that influences the final characteristics of gluten-free breads. In our formulas, the water content varies, also affecting the rheological properties of the dough. On the other hand, the effects of the ingredients can vary depending on other factors in the bread formulation, such as the specific ratios of ingredients [31]. The combined effects of psyllium husk fiber and xanthan gum could have influenced the rheological properties of the dough in such a way that the expected changes from HPMC addition were not observed. These ingredients may have increased the overall elasticity of the dough, counteracting the viscous behavior typically associated with HPMC. Mancebo et al. [17] observed that increasing HPMC in gluten-free bread formulations increased G′, but this effect of the hydrocolloid diminished until it became nonexistent as the amount of water in the formula was increased. However, Mancebo et al. [17] also reported an increase in Tan δ, an effect that was not observed in our study, possibly due to the presence of xanthan gum in our formulation.

The increase in psyllium in formula F2 significantly reduced the loss modulus (G″) compared to the other formulations, which also resulted in a decrease in the loss tangent, Tan δ. This effect was similar to the findings reported by Gao et al. [3] and Mancebo et al. [17], who documented a decrease in Tan δ with increased amounts of psyllium.

The elimination of xanthan gum in the formulation resulted in significantly lower G′ values compared to the other formulations. However, no significant differences were observed in the G″ values compared to the control formula, which led to a significantly higher Tan δ value than in the rest of the formulations. This can be attributed to the contribution of xanthan gum to the dough’s elasticity [26]; its absence results in less elastic dough.

### 3.2. Dough Extrusion

**Figure 2:**
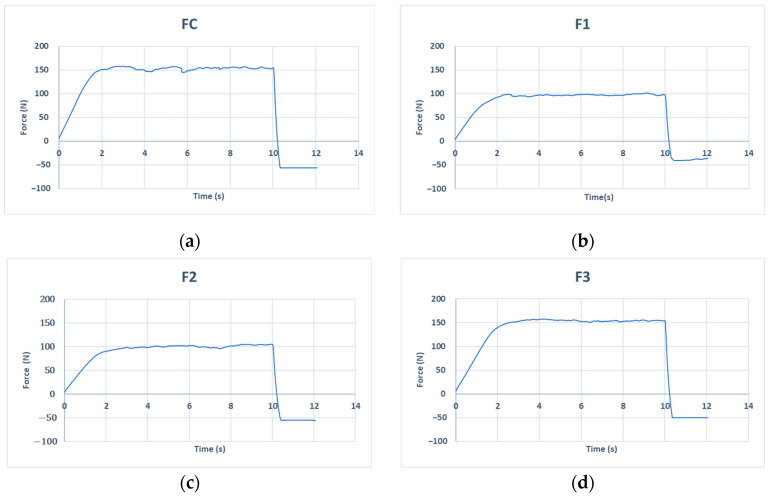
Extrusion force. (**a**) FC: control formula; (**b**) F1: HPMC increase; (**c**) F2: psyllium increase; (**d**) F3: xanthan gum removal.

Typical force-versus-time curves obtained during forward extrusion measurements are shown in Figure 2. These curves reflect the different stages of extrusion. The piston pressed onto the sample, compressing it to pack it more tightly [32]. In this stage, between 0 and 2 s, the force increased rapidly and extrusion occurred. Once the force reached a peak, a plateau was observed at 4 s. As seen in Figure 2, the control formula (FC) and F3 exhibit similar behavior in terms of extrusion force. Similarly, F1 and F2 also display comparable behaviors.

In Table 3, a significant reduction in both average force and area is highlighted in formulations F1 and F2, where the amount of HPMC and psyllium was increased, respectively, compared to the control formulation (FC). This increase in the concentration of these two hydrocolloids reduced the firmness of the dough and facilitated its extrusion.

In the studies conducted by Belorio et al. [22], the firmness of the pastes resulting from the RVA analysis of a corn starch mixture with different percentages of psyllium or xanthan was analyzed. It was observed that an increase in psyllium or xanthan gum reduced the firmness of the paste. In the work reported by Sciarini et al. [24], the effects of various hydrocolloids (carrageenan, alginate, xanthan gum, carboxymethylcellulose, and gelatin) on a gluten-free flour mixture were explored. During extrusion tests, it was noted that the dough containing xanthan gum required greater force compared to the control formula and other formulations containing different hydrocolloids. This phenomenon was attributed to the high-molecular-weight molecules of xanthan gum, which form complex aggregates through hydrogen bonding and polymer entanglements, resulting in high Newtonian viscosity at low shear rates [24].

However, in our study, F3, although the extrusion force was reduced, did not have a significant effect compared to the control formulation (FC). It is possible that the removal of xanthan gum combined with water reduction resulted in characteristics similar to FC. Higher amounts of xanthan gum in extrusion tests resulted in doughs with greater firmness [33].

### 3.3. Physicochemical Analysis

Table 4 displays the results for the four formulations regarding moisture content and water activity in the dough, crust, and crumb. The results of the pH measurements in the crumb are also shown.

Hydrocolloids are added to bakery products to improve their shelf life by maintaining moisture content and delaying staling [34]. Looking at the moisture content results (x_w_), as shown in Table 4, it can be seen that the moisture content of the dough is directly related to the amount of water that each formulation contains (Table 1). F2 has the highest water content in the recipe and consequently, the highest moisture percentage in the dough, while F3 contains the least water and the lowest moisture percentage.

Observing the moisture content results for the crust, although there are no significant differences among the formulations, the same pattern observed in the dough is repeated. F2 has the highest moisture content, and F3 has the lowest moisture content in the crust.

In the moisture analysis results for the crumb, no significant differences were observed compared to the control formula (FC). F2 continues to show the highest moisture content, while F3 has the lowest. Research conducted by Fratelli et al. [35] used various proportions of water and psyllium, finding that an increase in water content in the formula increased crumb moisture. This study also revealed that higher water content in the formulations led to an increase in the specific volume of the bread and a loss of weight during baking. In another study, Sabanis and Tzia [36] found that bread containing 2 g/100 g of hydrocolloid showed the highest moisture values in the crumb and crust after one day, due to the higher amount of water used in preparing these samples.

Water activity (a_w_) values (Table 4) showed that formulation F1, where the amount of HPMC was increased, presented no significant differences in the a_w_ of the dough and crust compared to the control formulation (FC). However, the a_w_ of the crumb in F1 was significantly higher compared to the FC. In formulation F2, where the amount of psyllium was increased, there was a significant increase in the a_w_ of both the crumb and crust compared to the FC. The increase in aw in formulations F1 and F2 is likely related to an increase in moisture in these formulas. In the study conducted by Encina-Zelada et al. [33], an increase in a_w_ in the crumb was observed when the amount of water in the recipe was increased. However, F3, where xanthan gum was removed, showed lower a_w_ values despite having lower moisture in the dough. This could be due to the effect of xanthan gum in reducing a_w_. This phenomenon was also observed by Encina-Zelada et al. [33], who reported a decrease in a_w_ in the crumb with an increase in the amount of xanthan.

The values of pH (Table 4) indicate that the crumb of the control formulation (FC) recorded a significantly lower pH. The rest of the formulations had a higher pH than the FC. F3, where xanthan gum was removed, turned out to be the formulation with a significantly higher pH compared to the others. In the study by Encina-Zelada et al. [33], it was observed that an increase in xanthan gum affected decreasing pH. On the other hand, Ziemichód et al. [37] demonstrated a decrease in pH when incorporating ground psyllium seeds into the control formulation, although this effect was greater when the seed was whole.

### 3.4. Color Analysis

Color is an important characteristic of baked products because it contributes to consumer preference [36]. Table 5 displays the means and standard deviations of the results acquired during the color characterization process.

The color analysis results presented in Table 5 indicate that the increase in HPMC in formulation F1 did not show significant differences in the color of the dough, crust, or crumb compared to the control formulation (FC), except in the b* parameter of the dough. Although this parameter was significantly higher than in the FC, it did not increase as much as in the rest of the formulations. Other studies have observed varied results; for example, a decrease in L* values was reported with an increase in HPMC in gluten-free breads. Meanwhile, Sabanis and Tzia [36] found a reduction in the luminosity of the crust and an increase in the crumb when adding HPMC to different types of flour. In this same study, it was noted that the addition of HPMC affected the a* and b* values depending on the type of flour used. In the works of Kim and Yokoyama [23], HPMC reached the highest values of a* and b* when added to corn starch, while its addition to rice flour did not produce this effect.

In formulation F2, where the amount of psyllium was increased, significant differences in color were observed compared to the control formulation (FC). The dough of F2 showed lower L* values and higher a* and b* values. This effect is consistent with the findings of Belorio et al. [22], where RVA analysis gels containing corn starch and psyllium displayed a darker color and higher a* and b* values than those obtained with starch and xanthan gum. Regarding the results in the crust and crumb, the increase in psyllium did not affect the L* value, but it did decrease the a* and b* values, with the a* value in the crumb being significantly higher than in other formulations, suggesting an increase in the reddish tone due to the increase in psyllium in the formulation. Studies conducted by Gao et al. [3] showed that increasing the amount of psyllium in a formulation containing buckwheat flour decreased the L* value in the crust, while a* and b* values increased; in the crumb, the L*, a*, and b* values also increased. Ziemichód et al. [37] indicates that adding ground psyllium seeds to a gluten-free bread formulation containing rice flour resulted in a reduction in luminosity and a very significant increase in a*, as well as a decrease in b* in the bread crumb.

F3, where xanthan gum was removed, showed differences compared to FC in the color of the dough, with increases in L*, a*, and b* values, whereas in the crust, L* and b* values decreased. However, the most significant differences were observed in the color of the crumb, where there was a significant reduction in a* and b* values, with a* experiencing the greatest decrease due to the removal of xanthan gum. Therefore, we can highlight that the removal of xanthan gum from the formula resulted in a reduction in the reddish tones of the crumb. Nonetheless, no changes were observed in the L* value in the bread crumb. In studies by Belorio et al. [31], it was observed that breads made with xanthan gum had the highest a* values in preparations containing rice flour and the lowest b* values among those made with corn starch. Tebben et al. [38] added xanthan gum to whole wheat flour and observed that in the crust color, it did not affect the L*, but slightly increased a* and b*, providing more red and yellow tones.

Regarding the color differences, ∆E indicates that only color differences are perceptible in the bread crust for the evaluated formula. It can be considered that to be perceptible by the human eye, ∆E values must be higher than three [39]. In our study, this value is only exceeded in the crust and in a very small way.

### 3.5. Textural Characterization

Starch retrogradation is recognized as one of the main factors contributing to bread staling. This process is defined as the reassociation of dispersed amylose and/or amylopectin molecules through hydrogen bonds, forming three-dimensional network structures [40]. In the research by Roman et al. [41], it was observed that the length of amylose plays a more important textural role than its content in the production of gluten-free products. The hardening of the crumb, which is characterized by an increase in its hardness over time, is usually accompanied by a loss of cohesion, elasticity, and resilience [42]. Moreover, texture is crucial in the sensory experience of the consumer, as changes in this parameter can significantly influence the ease and satisfaction during mastication [43].

Table 6 presents the results of the TPA analysis of the crumb for different formulations. In this table, significantly lower hardness can be observed in F3, which eliminated xanthan gum, compared to the other formulations. In the study by Crocket et al. [44], it was observed that increasing the amount of xanthan gum decreased the specific volume of the bread as a result of increased elasticity and decreased extensibility of the dough, which led to an increase in crumb hardness. This same effect was observed by other authors [21,26,45]. However, Sciarini et al. [24] found that adding xanthan gum reduced the hardness of the crumb and increased the specific volume of the bread. Therefore, it is possible that the effect of xanthan gum depends on the mix of ingredients used. Mancebo et al. [17] observed a significant inverse relationship at 99% between the specific volume of the bread and the initial firmness, using hydrocolloids such as HPMC and psyllium in their formulations. This leads us to think that possibly F3 might also have a greater specific volume as a result of the elimination of xanthan gum.

HPMC and psyllium husk fiber have been reported to play significant roles in crumb softening in various studies [18,21,46,47]. HPMC has been found to have a noticeable effect on dough rheological properties, leading to a more strengthened dough. This could potentially influence the texture of the bread crumb. HPMC contributes to the moistness of the bread. The moisture loss of bread containing HPMC was slower compared to other breads. This could potentially result in a softer crumb texture due to higher moisture content. Psyllium husk fiber has been reported to reduce bread hardness and increase its cohesiveness and resilience, thus lowering staling. This could result in a softer crumb texture. The addition of psyllium husk fiber can also contribute to an increased moisture content of the crumb. This effect could potentially contribute to a softer crumb texture. In our study, and in a similar way for rheological parameters, the increase in HPMC (F1) or psyllium (F2) does not imply a significant reduction in hardness or other textural properties. These results can be associated, as mentioned before, with the combined effects of HPMC, psyllium husk fiber, and xanthan gum in formulations and the different water content of the dough that result in similar rheological properties of dough, and as a consequence, similar textural properties of crumbs.

Gumminess is defined as the product of hardness and cohesiveness. Formulation F2, in which the amount of psyllium was increased, showed the highest gumminess value. This can be attributed to the fact that an increase in psyllium enhances hardness compared to other hydrocolloids like HPMC [13], but it also has an effect on increasing cohesiveness [37]. High cohesiveness values are desirable because they help form a bolus during mastication instead of disintegrating, while low cohesiveness is associated with a greater susceptibility of the bread to crumble [48]. Across all formulations, no differences in crumb cohesiveness were observed compared to the control formulation (FC), although the increase in HPMC in F1 decreased its value compared to F2, which, as mentioned earlier, increased with the addition of psyllium.

Adhesiveness is defined as the work required to separate a material from a surface. As shown in Table 6, the crumb of F3 is less adhesive. This effect was observed by Enzina-Zelada et al. [33], where the decrease in xanthan gum reduced the adhesiveness of the crumb.

It is important to note that no significant differences were observed in the texture parameters analyzed between the control formulation (FC) and F1, which contained an increase in HPMC. In the study by Mancebo et al. [17], where different proportions of HPMC, psyllium, and water were examined, no significant changes in volume or crumb hardness were detected with the addition of HPMC (2–4%) at any of the hydration levels studied. Previous studies [19,34,49] have shown that the presence of HPMC in formulations with wheat flour improves bread texture by reducing crumb hardness. This same effect was observed by Belorio and Gómez [31] when adding HPMC to rice flour or corn starch.

### 3.6. Image Analysis

The crumb structure of bread is an important quality criterion used in research laboratories and commercial baking to judge the quality of bread along with flavor, crumb color, and the physical texture of the crumb [50].

**Figure 3:**
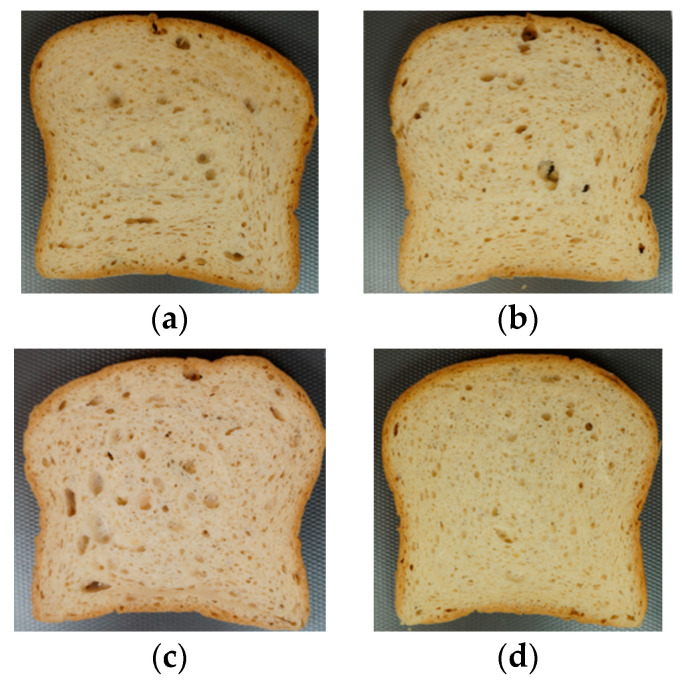
Slice images. (**a**) FC: control formula; (**b**) F1: HPMC increase; (**c**) F2: psyllium increase; (**d**) F3: xanthan gum removal.

In Figure 3, images of slices from the four formulations under study are shown. It can be observed that F2, with the increased psyllium, had a more irregular crumb and larger cell size, while F3, where xanthan gum was removed, had a more uniform crumb with smaller cell size.

Table 7 presents the results for the number of cells and the average cell area size obtained from the image analysis of samples. These results show that formulation F2, which contained a higher amount of psyllium, displayed a significantly lower number of cells compared to the other formulations, which may be due to the larger cell size, as depicted in Figure 3. However, there were no significant differences regarding the average cell area size, similar to the rest of the formulations. This is likely because F2 had cells with an area exceeding 10.00 mm^2^, which was the upper limit for the cells to be considered by the software.

F3, which had xanthan gum removed, recorded the highest value in the number of cells, and as seen in the image of the crumb in Figure 3, the slice exhibited a fine and regular structure. Generally, a large number of small, uniform, and thin-walled cells is preferred because the resulting texture is softer and more elastic compared to cells that are large and thick-walled [38].

In the study conducted by Encina-Zelada et al. [33], it was observed that reducing xanthan gum in formulations resulted in crumbs with larger cell sizes and a lower number of cells. However, increasing the amount of xanthan gum or decreasing the amount of water produced the opposite effect: cell size decreased and the number of cells increased. Peressini et al. [51] observed that increasing xanthan gum reduced the diameter of gas cells, using xanthan gum as the sole hydrocolloid in the formula. Tebben et al. [38] noted that a gradual increase in xanthan from 0.1% to 1% in a recipe with whole wheat flour increased the number of cells. The increase in HPMC in formula F1 showed no significant differences compared to FC. These results are similar to those found by Barcenas et al. [19], where the addition of HPMC to the formula did not significantly affect the number and size of the cells.

## 4. Conclusions

According to this study, significant differences were found among the tested formulations. Formulation F1, with a higher HPMC content than the control formulation (FC), required less force during extrusion. This suggests that an increase in HPMC could facilitate more precise dosing in industrial lines utilizing extrusion. However, this increase did not affect the elasticity, viscosity, or bread quality. Consequently, increasing the HPMC content could raise recipe costs without improving the analyzed parameters.

Incorporating psyllium into formulation F2 resulted in a decrease in the Tan δ value in the dough rheology. Therefore, it can be inferred that the increase in psyllium improves dough elasticity, which could complicate dough processing in lines using sheeting for dough piece formation. Additionally, an increase in psyllium led to a rise in the a* color value of the crumb, resulting in redder bread slices. Furthermore, this increase in psyllium was associated with higher crumb gumminess and crust hardness, as per the puncture analysis.

Finally, formulation F3 involved the exclusion of xanthan gum. Consequently, a notably different dough rheology was observed, with a lower G′ value and a higher Tan δ value compared to the control formulation (FC). This suggests that the dough is less elastic, facilitating its sheeting in gluten-free dough production lines. Regarding extrusion force, no significant differences were observed between formulation F3 and the control (FC). The bread analysis revealed that formulation F3 produced bread with a less firm and gummier crumb, as well as a less adherent crumb. Additionally, a decrease in the a* color value of the crumb was noted, resulting in a less reddish crumb.

In summary, excluding xanthan gum in gluten-free bread formulations containing psyllium and HPMC could potentially improve both dough processing in production lines and the characteristics of the final product.

Our research presented several limitations that should be considered when interpreting the results. The controlled experimental conditions do not fully replicate industrial conditions, which could influence the practical applicability of the results. Additionally, the subjective visual assessment used to determine the amount of water may introduce variability in dough consistency.

Future research could focus on replicating this study under industrial conditions to evaluate the practical applicability of the results. Further investigations could explore the impact of other hydrocolloids or combinations of ingredients on the quality of gluten-free bread. Finally, using more objective techniques to measure the amount of water could improve the consistency and reproducibility of the results.

## Figures and Tables

**Table 1:** Composition of the formulations in this study (g/100 g of flour and starch).

Ingredient	Xanthan Gum (g)	HPMC (g)	Psyllium (g)	Water (g)
Formula				
FC	3.2	3.6	9.8	159.7
F1	3.2	4.4	9.8	164.0
F2	3.2	3.6	13.2	173.6
F3	0	3.6	9.8	148.5

FC: control formula. F1: HPMC increase. F2: psyllium increase. F3: xanthan gum removal.

**Table 2:** Mean values ± standard deviations of rheological properties (G′, G″, Tan δ) of the different formulations obtained through frequency sweeps (5 Hz).

Formula	G″ Storage Modulus (Pa)	G″ Loss Modulus (Pa)	Tan δ (G″/G′)
FC	11,410 ± 310 ^b^	3431 ± 60 ^b^	0.301 ± 0.003 ^b^
F1	11,533 ± 25 ^b^	3517 ± 41 ^b^	0.305 ± 0.004 ^b^
F2	11,210 ± 236 ^b^	3207 ± 106 ^a^	0.286 ± 0.005 ^a^
F3	9305 ± 296 ^a^	3507 ± 179 ^b^	0.377 ± 0.007 ^c^

The same letter in superscript within the column indicates homogeneous groups established by ANOVA (*p* < 0.05). FC: control formula. F1: HPMC increase. F2: psyllium increase. F3: xanthan gum removal.

**Table 3:** Mean values ± standard deviations of analysis of extrusions.

Formula	Force (N)	Area (N·s)
FC	135 ± 47 ^c^	949 ± 57 ^c^
F1	88 ± 8 ^a^	621 ± 28 ^a^
F2	101 ± 4 ^ab^	708 ± 211 ^ab^
F3	129 ± 30 ^bc^	904 ± 331 ^bc^

The same letter in superscript within the column indicates homogeneous groups established by ANOVA (*p* < 0.05). FC: control formula. F1: HPMC increase. F2: psyllium increase. F3: xanthan gum removal.

**Table 4:** Mean values ± standard deviation of moisture content x_w_ (%) and water activity (a_w_) in the dough, crust, and crumb of the bread, and pH analysis in the crumb.

Formula	Dough x_w_ (%)	Dough a_w_	Crust x_w_ (%)	Crust a_w_	Crumb x_w_ (%)	Crumb a_w_	Crumb pH
FC	50.7 ± 1.5 ^ab^	0.911 ± 0.006 ^ab^	24.8 ± 3.4 ^a^	0.908 ± 0.016 ^b^	45.8 ± 1.7 ^ab^	0.937 ± 0.003 ^a^	5.13 ± 0.07 ^a^
F1	51.9 ± 0.1 ^bc^	0.909 ± 0.004 ^ab^	26.5 ± 2.7 ^a^	0.899 ± 0.034 ^b^	45.5 ± 1.4 ^ab^	0.940 ± 0.004 ^b^	5.21 ± 0.16 ^b^
F2	53.0 ± 0.1 ^c^	0.899 ± 0.013 ^a^	29.5 ± 6.3 ^a^	0.921 ± 0.018 ^c^	47.1 ± 1.7 ^b^	0.944 ± 0.002 ^c^	5.22 ± 0.07 ^b^
F3	49.8 ± 0.2 ^a^	0.921 ± 0.014 ^b^	24.6 ± 7.3 ^a^	0.871 ± 0.028 ^a^	44.1 ± 2.6 ^a^	0.939 ± 0.007 ^ab^	5.32 ± 0.05 ^c^

The same letter in superscript within the column indicates homogeneous groups established by ANOVA (*p* < 0.05). FC: control formula. F1: HPMC increase. F2: psyllium increase. F3: xanthan gum removal.

**Table 5:** Mean values ± standard deviations of color coordinates (L*, a*, b*, and ∆E) in the dough, crust, and crumb of the bread.

	FC	F1	F2	F3
L* dough	75.4 ± 0.4 ^b^	75.4 ± 0.2 ^b^	74.7 ± 0.3 ^a^	76.1 ± 0.3 ^c^
a* dough	0.30 ± 0.06 ^a^	0.33 ± 0.06 ^a^	0.48 ± 0.07 ^b^	0.56 ± 0.07 ^c^
b* dough	9.64 ± 0.15 ^a^	9.87 ± 0.13 ^b^	10.06 ± 0.15 ^c^	10.35 ± 0.25 ^d^
∆E dough		0.46 ± 0.26 ^a^	0.83 ± 0.32 ^b^	1.19 ± 0.30 ^c^
L* crumb	65 ± 2 ^a^	65 ± 2 ^a^	64 ± 3 ^a^	64 ± 3 ^a^
a* crumb	1.1 ± 0.3 ^b^	1.1 ± 0.3 ^b^	1.5 ± 0.4 ^c^	0.9 ± 0.4 ^a^
b* crumb	11.5 ± 0.9 ^c^	11.2 ± 0.8 ^bc^	11.0 ± 0.9 ^ab^	10.8 ± 1 ^a^
∆E crumb		2.6 ± 1.6 ^a^	2.6 ± 1.5 ^a^	3.1 ± 1.8 ^a^
L* crust	54 ± 3 ^b^	53 ± 3 ^b^	54 ± 3 ^b^	51 ± 2 ^a^
a* crust	9.6 ± 1.1 ^b^	9.3 ± 0.9 ^b^	8.7 ± 1.6 ^a^	9.6 ± 0.8 ^b^
b* crust	20.8 ± 2.5 ^b^	20.4 ± 1.9 ^b^	18.8 ± 2.8 ^a^	19.4 ± 2.4 ^a^
∆E crust		4.4 ± 1.6 ^a^	4.6 ± 1.5 ^ab^	5.1 ± 1.8 ^b^

L*: Lightening, a*: red-green; b*: yellow-blue. The same letter in superscript within rows indicates homogeneous groups established by ANOVA (*p* < 0.05). FC: control formula. F1: HPMC increase. F2: psyllium increase. F3: xanthan gum removal.

**Table 6:** Mean ± standard deviation of the texture profile analysis (TPA) of the crumbs in this study.

Formula	Hardness (N)	Adhesiveness (N·s)	Cohesiveness	Gumminess (N)
FC	58.9 ± 7.9 ^b^	−0.04 ± 0.03 ^a^	0.60 ± 0.02 ^ab^	35.3 ± 4.9 ^b^
F1	60.6 ± 10.2 ^b^	−0.04 ± 0.03 ^a^	0.59 ± 0.13 ^a^	35.5 ± 9.6 ^b^
F2	64.7 ± 13.8 ^b^	−0.05 ± 0.02 ^a^	0.63 ± 0.05 ^b^	40.7 ± 7.5 ^c^
F3	46.3 ± 11.6 ^a^	−0.02 ± 0.02 ^b^	0.60 ± 0.04 ^ab^	27.8 ± 7.60 ^a^

The same letter in superscript within the column indicates homogeneous groups established by ANOVA (*p* < 0.05). FC: control formula. F1: HPMC increase. F2: psyllium increase. F3: xanthan gum removal.

**Table 7:** Mean values ± standard deviations of number of cells (cells/cm^2^) and mean cell area (cm^2^).

Formula	Number of Cells (Cells/cm^2^)	Mean Cell Size (cm^2^)
FC	11 ± 2 ^b^	0.0083 ± 0.0004 ^a^
F1	11 ± 2 ^b^	0.0083 ± 0.0007 ^a^
F2	8 ± 4 ^a^	0.0084 ± 0.0007 ^a^
F3	12 ± 1 ^c^	0.0082 ± 0.0007 ^a^

The same letter in superscript within the column indicates homogeneous groups established by ANOVA (*p* < 0.05). FC: control formula. F1: HPMC increase. F2: psyllium increase. F3: xanthan gum removal.

## Data Availability

The data presented in this study are available on request from the corresponding author. The data are not publicly available due to privacy restrictions.
